# United States Veterinary Medical Association

**Published:** 1891-03

**Authors:** W. Horace Hoskins

**Affiliations:** Secretary


*United States Veterinary Medical Association.*—At a special meeting of the Comitia Minora of the United States Veterinary Medical Association, held at the Hotel Royal, New York City, on January 27, 1891, at 8 P. M., the followlng members were present: Drs. Coates, Huidekoper, T. B. Rayner, William Dougherty, Hoskins, James L. Robertson, R. A. McLean, and Winchester. Absent: Drs. Williams, Butler, and Lyford.

Chairman Coates stated the objects of the meeting and Secretary Hoskins offered the following resolution: "That we recommend to the Association that the meeting for 1893 be held in Chicago and that it be International in character." After some discussion an amendment was offered and accepted that a committee of three be appointed to propose the subjects for discussion, which was carried.

The place for the convention of 1891 was then considered, the Secretary reading appeals for Boston, Baltimore, and Washington, with a letter from Dr. Williams, one of the Western members of the committee and, after discussion, a motion was made that we meet in Washington, D. C., which was carried.

A motion was then offered and adopted that a committee of three be appointed to complete the necessary arrangements, and the Chairman announced the names of Drs. Huidekoper, Hoskins, and Dougherty, as the committee.

After the disposal of some other routine matters the meeting adjourned.

W. HORACE HOSKINS, *Secretary.*

